# Editorial: The Role of the Lymphatic System in Lipid and Energy Metabolism, and Immune Homeostasis During Obesity and Diabetes

**DOI:** 10.3389/fphys.2021.652461

**Published:** 2021-03-04

**Authors:** Vincenza Cifarelli, Hong Chen, Joshua P. Scallan

**Affiliations:** ^1^Department of Medicine, Center for Human Nutrition, Washington University School of Medicine, St. Louis, MO, United States; ^2^Vascular Biology Program, Harvard Medical School, Boston Children's Hospital and Department of Surgery, Boston, MA, United States; ^3^Department of Molecular Pharmacology and Physiology, Morsani College of Medicine, University of South Florida, Tampa, FL, United States

**Keywords:** lymphatic system, lacteal, obesity, Prox1, chylomicron, hypercholesterolemia, endothelial plasticity, chronic liver disease

The Research Topic “*The Role of the Lymphatic System in Lipid and Energy Metabolism, and Immune Homeostasis During Obesity and Diabetes”* summarizes the rapid development of the field of lymphatic vessel biology over the last two decades emphasizing its relevance to metabolism and metabolic dysregulated diseases. The Research Topic, available in *Frontiers in Physiology—Lipid and Fatty Acid Research*, comprises 11 Reviews of the current literature, one Original Research Article, one Perspective, and one Hypothesis and Theory by experts in the field.

The involvement of the lymphatic system in the transport and distribution of lipids has been known for centuries (Suy et al., 2016), despite receiving relatively modest attention due to the wrong belief of passive unregulated drainage of the lymph. Although much uncharted scientific territory remains to be explored in this still nascent field, the regulation of gut lymphatics is experiencing a surge in interest as the associated metabolic outcomes are now starting to be elucidated, and appreciated as potential targets to manage obesity and its complications (Oliver et al., 2020). The Research Topic includes Reviews that summarize regulation of lipid trafficking and absorption, and impact on metabolism by the intestinal lymphatic capillaries (lacteals) and collecting vessels. Xiao et al. discuss the emerging evidence that oral glucose and intestinal hormone glucagon-like peptide-2 (GLP-2) mobilize retained enteral lipid into the lymphatic system by two different mechanisms; (i) via glucose, by mobilizing cytoplasmic lipid droplets and (ii) via GLP-2, by targeting post-enterocyte secretory mechanisms. A second contribution by Zhou et al., discusses the importance of composition and properties (i.e., degree of hydration) of the matrix in the lamina propria in regulating chylomicron diffusion and their entry in the lacteals. Zhou et al. provide insights into the activation of mucosal mast cells by fat absorption and the associated metabolic consequences highlighting the link between chylomicron transport, mucosal mast cell activation, leaky gut, and the microbiome. In the *Hypothesis and Theory Article*, Nauli and Matin discuss a potential mechanism related to higher accumulation of abdominal visceral fat in males as compared to pre-menopausal women. The authors propose that the accumulation of abdominal visceral fat in men, a strong independent predictor of mortality, is due to the higher dietary fat uptake by abdominal visceral fat. During the post-prandial state, the larger chylomicrons observed in males congest the lamina propria and the low-pressure lymphatics, predisposing the chylomicron triglycerides to hydrolysis by lipoprotein lipase. The liberated fatty acids are then stored by the nearby abdominal visceral adipocytes, leading to the accumulation of abdominal visceral fat. These mechanisms perhaps explain the reason of bigger and higher production of chylomicrons in males.

The link between the lymphatic system, metabolic dysfunction, inflammation, and the metabolic syndrome is not completely understood and likely to involve multiple mechanisms and different organs. A growing body of evidence suggest that dysfunctional lymphatic vasculature might promote obesity by impairing adipogenesis and promoting inflammation. This is somehow not surprising given the adipogenic properties of lymph (Harvey et al., 2005) and the role of inflammation in adipose tissue expansion (Crewe et al., 2017). These concepts are explored in the review by Kataru et al., which provides an overview of how metabolic disease and inflammation interact to alter lymphatic function in the context of obesity, hypercholesterolemia, and diabetes. Particular attention is given to the role of inflammatory cytokines and immune cells with respect to various aspects of lymphatic function, including lymph transport, junctional integrity, permeability, and contractile function. A study by Chakraborty et al. demonstrates that overexpression of the inflammatory growth factor, VEGF-D, in adipose tissue induces the growth of new lymphatic vessels by lymphvasculogenesis and investigates how inflammatory cytokines and immune cell populations change over time. Ho and Srinivasan and Norden and Kume discuss the bi-directional relationship between obesity and the lymphatic vasculature, reviewing mechanisms and signaling pathways of dysfunctional lymphatic vasculature in adipose tissue expansion and obesity-associated inflammation. The reciprocal correlation between obesity and lymphatic dysfunction is further discussed by Ho and Srinivasan which summarizes clinical correlations and differences between obesity, lymphedema, and lipedema, given that these three diseases all present excessive adiposity, in particular in the lower limbs, and dysfunctional lymphatic vessels. Pharmacological interventions as well as behavioral and lifestyle changes that aim to improve lymphatic function are also reviewed (Norden and Kume). Mouse models of obesity with defective lymphatic function are summarized in Norden and Kume.

The Research Topic reviews the contribution of lymphatic biology in metabolically dysregulated diseases, including type 2 diabetes, atherosclerosis, myocardial infarction, and chronic liver disease. Jiang et al. review how insulin resistance, diabetes, atherosclerosis, and myocardial infarction interact with the lymphatic vasculature. Lymphatic vessels are also located in the adventitial and periadventitial region of arterial walls and exert an important function in mobilizing efficiently cholesterol out of the vessel wall. The role of the lymphatic system in the onset and pathogenesis of atherosclerosis is summarized in Milasan et al., which also discuss the contribution and the diversity of lymph extracellular vesicles (exosomes, microvesicles, and apoptotic bodies) within the lymphatic function and progression of atherosclerosis. Another critical metabolic organ where lymphatic vessels are currently understudied is the liver. Burchill et al. review the inherent difficulties of using lymphatic markers to identify lymphatic vessels in the liver, where hepatic cells express many of the same proteins. The focus of the review, however, is summarizing newly identified roles for lymphatic endothelium in non-alcoholic steatohepatitis and hepatitis C infection.

Lymphatic endothelial cell (LEC) junctions are crucial for the maintenance of vessel integrity and proper lymphatic vascular function. Zhang et al. provide a comprehensive review of the molecular regulation of LEC junctions during development, and diseases namely obesity and inflammation-associated conditions. The authors provide a comprehensive summary of lymphatic vascular phenotypes following genetic deletion of junctional proteins.

Vascular diversity and endothelial plasticity are reviewed by Pawlak and Caron. The authors review five highly specialized hybrid vessel beds that adopt partial lymphatic programing for their specialized vascular functions namely (i) the high endothelial venules of secondary lymphoid organs, (ii) the liver sinusoid, (iii) the Schlemm's canal of the eye, (iv) the renal ascending vasa recta, and (v) the remodeled placental spiral artery. The authors summarize the morphology and endothelial expression pattern of these vessels and interrogate their specialized functions within the broader blood and lymphatic vascular systems (Pawlak and Caron). Finally, Azhar et al. discuss the current knowledge on the pathophysiology of lymphedema, a chronic and progressive disease arising from impaired lymphatic drainage, which causes accumulation of interstitial fluid and results in tissue swelling (Rockson, 2001). Authors review lymphedema diagnosis, assessment and available treatments and further discuss the cellular and molecular mechanisms involved in the expansion and remodeling of adipose tissue, as well as fibrosis and collagen accumulation occurring during lymphedema, listing animal models of lymphedema available for pre-clinical studies.

In conclusion, our understanding of the regulation of the lymphatic system in contributing to tissue remodeling, inflammation, and metabolic outcome during disease has greatly improved in the last two decades. However, more translational studies are needed to pave the way for novel therapeutic approaches to treat metabolically dysregulated diseases, including type 2 diabetes, atherosclerosis, myocardial infarction, and chronic liver disease, as well as lymphedema and lipedema.

## Author Contributions

All authors wrote and revised the manuscript. All authors contributed to the article and approved the submitted version.

## Conflict of Interest

The authors declare that the research was conducted in the absence of any commercial or financial relationships that could be construed as a potential conflict of interest.
